# Reducing work disability in Ankylosing Spondylitis – development of a work instability scale for AS

**DOI:** 10.1186/1471-2474-10-68

**Published:** 2009-06-16

**Authors:** Gill Gilworth, Paul Emery, Nick Barkham, M Glyn Smyth, Philip Helliwell, Alan Tennant

**Affiliations:** 1 Department of Rehabilitation Medicine, Section of Musculoskeletal Disease, Leeds Institute of Molecular Medicine, University of Leeds, Leeds, UK; 2 Section of Musculoskeletal Disease, Leeds Institute of Molecular Medicine, University of Leeds, Leeds, UK; 3 Work Fit Occupational Physiotherapy and Ergonomics Services Ltd, Leeds, UK; 4 Section of Musculoskeletal Disease, Leeds Institute of Molecular Medicine, University of Leeds, Leeds, UK; 5 Bradford Teaching Hospitals NHS Foundation Trust, Bradford, UK

## Abstract

**Background:**

The Work Instability Scale for Rheumatoid Arthritis (RA-WIS) is established and is used by physicians to identify patients at risk of job loss for rapid intervention. The study objective was to explore the concept of Work Instability (a mismatch between an individual's abilities and job demands) in Ankylosing Spondylitis (AS) and develop a Work Instability Scale specific to this population.

**Methods:**

New items generated from qualitative interviews were combined with items from the RA-WIS to form a draft AS-WIS. Rasch analysis was used to examine the scaling properties of the AS-WIS using data generated through a postal survey. The scale was validated against a gold standard of expert assessment, a test-retest survey examined reliability.

**Results:**

Fifty-seven participants who were in work returned the postal survey. Of the original 55 items 38 were shown to fit the Rasch model (χ^2 ^37.5; df 38; p 0.494) and free of bias for gender and disease duration. Following analysis for discrimination against the gold standard assessments 20 items remained with good fit to the model (χ^2 ^24.8; df 20; p 0.21). Test-retest reliability was 0.94.

**Conclusion:**

The AS-WIS is a self-administered scale which meets the stringent requirements of modern measurement. Used as a screening tool it can identify those experiencing a mismatch at work who are at risk of job retention problems and work disability. Work instability is emerging as an important indication for the use of biologics, thus the AS-WIS has the potential to become an important outcome measure.

## Background

Ankylosing Spondylitis (AS) is a chronic inflammatory disease of joints and entheses, typically affecting patients in their 20s when they are establishing their careers. Although estimates for Work Disability (WD) in AS vary [1,2] it is clear that absence from work and problems with job retention due to AS are significant. AS has been reported as the reason some patients have to change their occupation, reduce their hours of work, or find their career progression limited [3].

The period prior to WD is one of Work Instability (WI), defined as the consequences of a mismatch between a person's functional ability and the demands of their job, potentially threatening continuing employment if not addressed [4]. WI has attracted less attention than WD in the literature, however as a clinical concept it may be a key factor in the management of individuals with rheumatological conditions that occur during the working years. Data from the UK suggests that of those AS patients of working age, 50% have lost their job due to the activity of their disease, and of those in work 50% are work unstable, at moderate or high risk of job retention problems [5]. If WI is recognised early it may be possible to reduce or eliminate the risk of work disability by appropriate clinical or work place intervention.

There is no instrument currently available to measure WI in AS, the only way to identify clients who fulfil our definition of WI is through a comprehensive Vocational Assessment. This type of assessment is offered in the UK by Jobcentre Plus, Disability Service and includes analysis of work tasks and postures as well as medical history. Following such an assessment various interventions may be offered (for example specialist ergonomic equipment or adaptations to work practices). The degree of intervention required is dependent on the degree of WI.

A validated Work Instability Scale for rheumatoid arthritis (RA-WIS) has previously been developed [4]. From our work exploring WI in different conditions and for different occupations we have found that each group brings a unique component to WI as well as some common elements [6,7]. Thus there is a need to examine the factors relevant to WI separately, in each condition, to ensure that the scale captures the relevant elements specific to that disease. The objective of this study was to develop and validate a Work Instability Scale specific to AS.

## Methods

**Figure 1:**
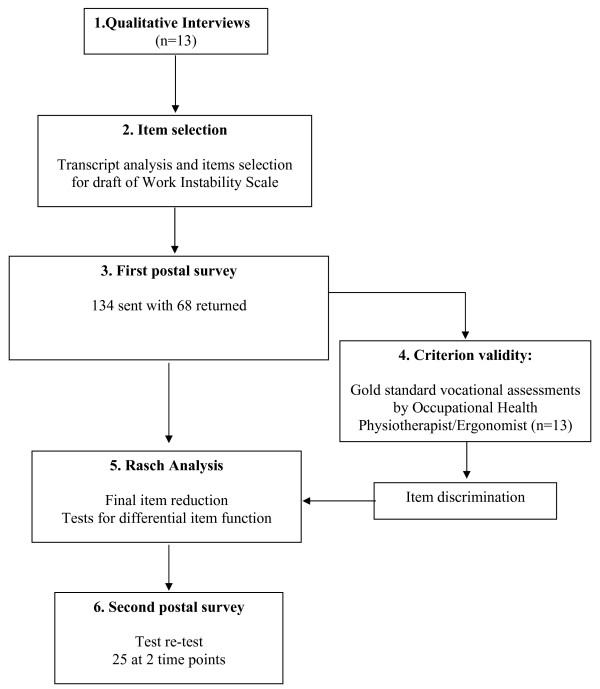
**Stages of the study**.

A similar staged methodology to that used in the development of the RA-WIS [4] was followed (see figure 1).

The entry criteria for the participants for all the stages of the study were as follows:

• Confirmed diagnosis of AS by modified New York criteria

• In paid work (but may be in current employment but "off sick" for less than 6 months in the current period); self-employed and part time workers are included.

• Aged between 18 – 60 years of age.

### Qualitative interviews

The aim of this stage of the study was to identify characteristics of WI unique to AS. Thirteen qualitative interviews were undertaken with participants attending rheumatology outpatients and fulfilling the above recruitment criteria. This satisfies current guidelines for minimum sample size to achieve saturation [8]. Selection of participants was based on a theoretical sample frame to ensure representation for age, gender and work type. Participants had a good range of occupations including sedentary workers (for example Administrator and a Town Planner who was mainly office based), light work (for example Hairdresser and Dental Technician) and manual workers (for example Plumber and Refuse Collector). The interviews took the form of informal conversations with the interviewer introducing areas for discussion using a topic list, main areas were as follows:

• Details of occupation at onset of AS, impact of AS then and now, adaptations required, need for part-time working, job security

• Employer – including disclosing diagnosis, attitude, flexibility of employment

• Access to and from work and within work

• Relationships (work and home)

The interviews were tape recorded in full and typed transcripts produced.

### Item selection

Thematic analysis of the interview transcripts was undertaken. Common issues relevant to WI were formulated into potential items for the draft AS-WIS. Where possible the exact words of the interviewees were used. New items generated from the interviews were combined with items thought to be relevant from the existing RA-WIS.

### First postal survey

A questionnaire booklet including demographic details, AS QoL [9] and the draft of the AS-WIS was sent to subjects with a confirmed diagnosis of AS, and of the relevant age. Subjects were all attending for treatment at the local rheumatology clinic in Leeds. Only a single questionnaire was distributed, without follow-up. The ASQoL was chosen as a comparator measure because it offers a disease-specific 'needs based' quality of life measure which would be expected to have a strong correlation with work instability, which is focused upon the construct of participation (i.e. the need amongst this age group to maintain work).

A filter question about employment status ensured that the AS-WIS was only completed by those currently working. The aims of this stage were to test the scaling properties of the draft WIS, to facilitate item reduction and to provide preliminary evidence of construct validity.

Criterion validity: comparison of draft instruments against a 'Gold Standard".

A sample of volunteers from the postal survey completed the draft AS-WIS a second time. On the same day they were assessed by an experienced Occupational Health Physiotherapist/Ergonomist who was blind to the responses on the draft WIS. A facilitator, who asked the patients to complete the questionnaire, also performed a cognitive debriefing at the same time. The expert allocated each participant a WI score between 0 and 4. This scoring system was devised and has been used successfully in the development of other WIS [4,6]. The draft AS-WIS questionnaire responses were then validated against the results of the gold standard assessments. Those items that were shown to discriminate across the levels of risk ascertained by the experts were retained for further analysis. Cut points for level of risk were then determined by those which maximised the sensitivity and specificity of the screening questionnaire for concordance with the expert judgement.

### Rasch Analysis

The Rasch model [10] is the current standard for the development of unidimensional scales (e.g. of impairment or quality of life) delivering metric quality outcomes in health care [11]. Briefly, data collected from questionnaires which include items for a new (or existing) scale, which are intended to be summated into an overall score are tested against the expectations of this measurement model. The model defines how responses to items should be if measurement (at the metric level) is to be achieved. The response patterns achieved are tested against what is expected (a probabilistic form of Guttman scaling [12]), and a variety of fit statistics determine if this is the case [13].

Within the framework of Rasch measurement, the scale should also work in the same way, irrespective of which group is being assessed [14]. For example, in the case of WI, males or females should have the same probability of affirming an item if they have the same underlying level of WI. If for some reason one group did not display the same probability of affirming the item, then this item would be deemed to display differential item function (DIF), and would violate the requirement of unidimensionality [15]. Consequently, every item is checked for DIF by age and gender and, in the current study by time, to ensure stability in the test-retest sample. Finally, a rigorous check for unidimensionality is undertaken by identifying contrast sets of items on the principal first component of the residuals and testing if person estimates derived from these sets differ. The confidence interval for the proportion of individual t-tests showing a difference between estimates should overlap 5% if the scale is strictly unidimensional [16].

The sample size requirements for Rasch analysis are based upon the degree of precision required for estimates of item difficulty and person ability. For example, in most cases a sample size of 50 will give an item calibration within 1 logit with 99% confidence [17]. This varies according to how well the scale is targeted at the patient sample. Thus a well targeted sample of 108 will give an estimate to within 0.5 logits with 99% confidence. It is important to note that Rasch analysis does not require a 'representative' sample as item difficulty is estimated independently from the ability of persons taking the test. It is more important to have a uniform distribution of persons such that the degree of precision of item estimates is similar across the whole of the construct (i.e. work instability) being measured.

Data are fitted to the Rasch model using the RUMM2020 software [18].

### Test-retest postal survey

A sample of in-work patients were asked to complete the new draft of the WIS on two occasions, two weeks apart. These patients were attending for routine rheumatology clinic appointments in Bradford, a city in northern England adjacent to Leeds. This stage of the study was to assess the test-retest reliability of the scale, and to provide further evidence of its internal construct validity.

Ethical committee approval was granted by Leeds Teaching Hospitals NHS Trust Local Research Ethics Committee under a programme of work for 'Reducing Work Disability in common rheumatic conditions' [Ref CA03/035].

## Results

### Qualitative interviews

Analysis of the qualitative interview transcripts indicated many common themes with people working with Rheumatoid Arthritis including the importance of flexibility at work the impact of symptoms and the need to change or adapt work tasks because of impairments. Some impairment specific themes were reflected in potential items for the AS-WIS including those relating to mobility (for example '*getting around at work is hard for me', 'its painful walking'*).

### Item selection

Fifty five potential items were formed into a draft AS-WIS, 40 of these items were derived directly from the AS interview transcripts including 6 items identical to those on the RA-WIS. A further 15 items thought relevant to the AS population from the RA-WIS were included.

### First postal survey

Of 134 subjects included in this stage of the study, sixty-eight returned the survey (response rate 51%). The mean age of participants in this stage of the study was 41.5. years (SD 9.8; range 26–60). Almost three-quarters (73.7%) were male. Eleven of those responding were currently off sick for a period of more than 6 months so did not meet the entry criteria. There was a trend for those not working to be older, but this did not reach statistical significance (t = 1.90 p = 0.061). The mean duration of disease of the 57 respondents in work was 19.2 years (SD 8.9).

Initial Rasch analysis of the questionnaires from these 57 respondents showed 38 items free of bias for gender and disease duration and which fitted the Rasch model (chi square 37.5; df 38; p 0.494).

### Criterion validity: comparison of draft instruments against a 'Gold Standard'

Over half (56%) of those in work and completing the first postal questionnaire volunteered for a full vocational assessment. There was no significant difference between those who did and did not volunteer, for age, level of work instability, or quality of life (Mann-Whitney; p > 0.05). In the event, 13 subjects were able to take time off work and attend for assessment. Each was graded by the experts to a level of work instability, and then items were tested for their ability to discriminate across these levels. Following this analysis 20 items of the AS-WIS satisfied these criteria and display good fit to the Rasch model (chi square 32.2;df 20;p 0.04). The items displayed strict unidimensionality in the first postal survey data with just 3.6% of the t-tests out of range and still retained good representation of the major themes identified in the qualitative interviews.

Ten items on the final 20 item AS-WIS are common with items on the RA-WIS. For high levels of risk for work instability the sensitivity of the AS-WIS is 100% and specificity 82%. Nearly two-thirds of respondents (58%) were rated as low risk, just over a third (35%) at medium risk, and seven percent at high risk of job loss. Analysis of the AS-WIS and AS Quality of Life scores for the respondents in work showed a correlation of 0.84 (Spearman's rho) showing that 72% of the variation in Quality of Life of those is associated with their level of WI.

### Test-retest postal survey

One hundred and twenty two questionnaires were sent of which 52 were returned (43%), and of whom 35 (29%) were returned from people in work, and 25 (71%) returned the questionnaire on both occasions. Pooling the two data sets together no items displayed differential item functioning by time, supporting the invariance of the scale over repeated measurements. Again, given limitations of sample size, the scale showed good fit to Rasch model expectations (Chi-Square Interaction p = 0.21) and satisfied the test for unidimensionality (independent t-test 10.8% 95% CI 5–17%). Internal consistency reliability was 0.95 and test-retest reliability (Spearman) was 0.94.

### Combined data

**Figure 2:**
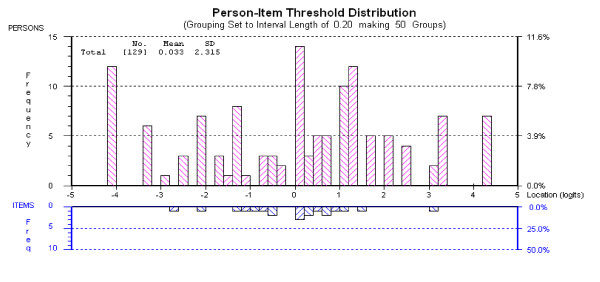
**Targeting of AS-WIS from pooled data**.

Given the relatively small sample sizes at each stage, the data from each survey was pooled to re-assess fit to the Rasch model, giving a sample of 123 cases with greater power to detect misfit and deviation from unidimensionality. The results confirmed the robustness of the scale, all individual items were shown to fit model expectations (Table 1), with overall fit (Chi-Square Interaction) showing a non-significant deviation from model expectation (p = 0.23) and strict unidimensionality (significant independent t-tests = 7.2% (CI: 3–11%). Reliability was high at 0.931, supporting the use of the scale at the individual level. The targeting of the scale was good, with a mean person estimate of 0.033 logits, suggesting the scale to be centered on the person distribution (Figure 2)

**Table 1:** Best and worse fitting items from the AS Work Instability Scale: Revalidation on First postal Questionnaire

**Item**	**Location**	**Residual**	**Chi** **Square**	Probability*
I have got to watch how much I do certain things at work	-0.548	-0.499	0.064	0.968
When I'm feeling tired all the time, works a grind	0.534	-0.479	0.595	0.743
When I'm in pain it affects my concentration	-2.146	-0.070	4.521	0.104
My condition is worse when I get stressed at work	0.183	1.824	7.581	0.023

* Bonferroni adjusted level 0.0025.

## Discussion

This study has produced a simple 20 item questionnaire to measure Work Instability in AS. It satisfies all the most stringent modern psychometric requirements for scale development, and demonstrates high levels of classical reliability. Two-fifths of those subjects who responded to the first postal questionnaire and were in work had medium or high levels of work instability, suggesting that sickness absence or job loss is an ever present threat to a substantial minority of those working with this condition. The high correlation with the disease specific quality of life scale (ASQoL) indicates how much, for those in work, the struggle to maintain work impacts upon quality of life.

There are a number of settings in which this scale will be potentially useful. In the outpatient clinic or therapy setting it will offer a simple, self-report, questionnaire which may be used as a screening tool to alert the clinician to the need for more detailed work assessment. It may also be useful in the employment/occupational health setting to assist in predicting/monitoring work suitability. WI is emerging as an important indication for the use of new therapeutics such as biologics, which are a relatively new type of drug designed to treat AS by targeting overactive cells in the body. Thus the AS-WIS also has the potential to become an important outcome measure in the research setting. Having identified the risk of job loss, a variety of interventions are possible to minimise the physical, financial and psychosocial impact of AS on the individual their family and their employer these may include early aggressive clinical treatment and/or preventative intervention in the workplace focused on reducing WI thus minimising sickness absence and risk of job loss.

We acknowledge that so far the instrument was developed upon and tested on a relatively small sample from the north of England; consequently the generalisability of the scale is currently unknown and predictive validity and responsiveness require further testing. The small sample sizes meant that the Rasch analysis had a relatively low degree of precision for model fit and the estimates of both item difficulty and person work instability. The power to detect DIF in the first postal questionnaire was also low, but we chose to examine this as anything showing DIF at this sample size would suggest substantive bias. The pooled data from the different studies improved the overall precision and gave greater confidence in the interpretation of the fit statistics, nevertheless a further study with a much larger sample (say > 200) is warranted before firm conclusions can be drawn. As different disease-and occupation-specific WI scales are developed, the common components of WI are being identified so that the different scales can be co-calibrated (by common item equating) to form a single item bank of work instability [19].

## Conclusion

A simple screening instrument for Work instability in AS has been developed which, despite some limitations due to small sample sizes, satisfies Rasch model expectations and has high reliability. Given that in the final scale half the items were shared with the original RA-WIS, the study provides evidence of the communality of some aspects of WI, but also the unique aspects brought to the concept by each diagnostic group. As an increasing number of common items are identified in different Work Instability Scales these can be used to establish an item bank to facilitate comparability across disease. It is hoped that in the future increased awareness of WI will help towards patients achieving their potential in work as well as other activities. With clinical cut points to identify the level of risk of job loss, the AS-WIS will help to fill a major gap in the currently available instruments for assessing the impact of AS on the working lives of patients.

Copies of the full AS-WIS scale with guidance notes and instructions for scoring are available from the Psychometric Laboratory for Health Sciences; contact v.e.lane@leeds.ac.uk.

## Competing interests

The authors declare that they have no competing interests.

## Authors' contributions

GG was the project leader contributing to study design, conducted the interviews, qualitative data analysis, postal surveys and prepared first full draft of manuscript. PE facilitated the study contributing to study design, assisted with the literature review, background work on study and assisted with preparation of manuscript. NB assisted with the literature review, background work on study and recruitment of study participants. MGS was the expert employment consultant on the project and completed the Gold Standard assessments. PH assisted with recruitment of study participants and project management. AT Facilitated study contributing to study design, completed Rasch analysis, wrote first draft of results section and assisted with preparation of the full manuscript and project management. All authors read and approved the final manuscript.

## Pre-publication history

The pre-publication history for this paper can be accessed here:


